# Dietary Predictors of Maternal Prenatal Blood Mercury Levels in the ALSPAC Birth Cohort Study

**DOI:** 10.1289/ehp.1206115

**Published:** 2013-06-28

**Authors:** Jean Golding, Colin D. Steer, Joseph R. Hibbeln, Pauline M. Emmett, Tony Lowery, Robert Jones

**Affiliations:** 1 Centre for Child and Adolescent Health, University of Bristol, Bristol, United Kingdom; 2 National Institute on Alcohol Abuse and Alcoholism (NIAAA), National Institutes of Health (NIH), Department of Health and Human Services, Bethesda, Maryland, USA; 3 National Oceanic and Atmospheric Administration (NOAA), National Marine Fisheries Service, National Seafood Inspection Laboratory, Pascagoula, Mississippi, USA; 4 Inorganic and Radiation Analytical Toxicology Branch, Centers for Disease Control and Prevention, Atlanta, Georgia, USA

## Abstract

Background: Very high levels of prenatal maternal mercury have adverse effects on the developing fetal brain. It has been suggested that all possible sources of mercury should be avoided. However, although seafood is a known source of mercury, little is known about other dietary components that contribute to the overall levels of blood mercury.

Objective: Our goal was to quantify the contribution of components of maternal diet to prenatal blood mercury level.

Methods: Whole blood samples and information on diet and sociodemographic factors were collected from pregnant women (*n* = 4,484) enrolled in the Avon Longitudinal Study of Parents and Children (ALSPAC). The blood samples were assayed for total mercury using inductively coupled plasma dynamic reaction cell mass spectrometry. Linear regression was used to estimate the relative contributions of 103 dietary variables and 6 sociodemographic characteristics to whole blood total mercury levels (TBM; untransformed and log-transformed) based on *R*^2^ values.

Results: We estimated that maternal diet accounted for 19.8% of the total variation in ln-TBM, with 44% of diet-associated variability (8.75% of the total variation) associated with seafood consumption (white fish, oily fish, and shellfish). Other dietary components positively associated with TBM included wine and herbal teas, and components with significant negative associations included white bread, meat pies or pasties, and french fries.

Conclusions: Although seafood is a source of dietary mercury, seafood appeared to explain a relatively small proportion of the variation in TBM in our UK study population. Our findings require confirmation, but suggest that limiting seafood intake during pregnancy may have a limited impact on prenatal blood mercury levels.

Citation: Golding J, Steer CD, Hibbeln JR, Emmett PM, Lowery T, Jones R. 2013. Dietary predictors of maternal prenatal blood mercury levels in the ALSPAC birth cohort study. Environ Health Perspect 121:1214–1218; http://dx.doi.org/10.1289/ehp.1206115

## Introduction

Concerns about adverse health effects of mercury exposure during fetal development stem in part from well-documented episodes of mass mercury poisoning from consuming food items grossly contaminated with mercury released into Minamata Bay in the 1950s and from consumption of wheat seed treated with mercury-based fungicides in the 1970s (D’ltri and D’ltri 1978). There have been reports of adverse reproductive effects of mercury, including infertility and miscarriage (Choy et al. 2002; Harada 1995), and of prenatal exposure being positively associated with blood pressure in children at 7 years of age (Sorensen et al. 1999), but the major concern has been the possible effect of prenatal mercury exposure on the brain of the developing fetus (Holmes et al. 2009). However, little research has been done on chronic exposures at low doses. In a cohort study conducted in the Faroe Islands (Grandjean et al. 1997), where seafood exposure was mainly from pilot whale consumption, 979 children were tested at 7 years of age and results compared with cord blood mercury levels; higher mercury levels were associated with subtle deficits in verbal development in language, attention, and memory. On the basis mostly of these findings, the U.S. National Research Council (NRC 2000) established a reference dose level of 5.8 μg/L of mercury in cord blood. Although advisories intended to reduce fetal exposure to mercury have concentrated on reducing maternal consumption of seafood (U.S. Food and Drug Administration 2004), adverse associations between prenatal maternal seafood consumption and childhood cognition have not been replicated by studies conducted in the Seychelles (Davidson et al. 1998, 2008, 2010), the United Kingdom (Hibbeln et al. 2007), Denmark (Oken et al. 2008), the Faroe Islands (Choi et al. 2008), and the United States (Lederman et al. 2008).

The primary goal of the present study is to evaluate the assumption that seafood consumption is a major contributor to maternal blood levels of mercury. We take advantage of a major British birth cohort survey (ALSPAC; Avon Longitudinal Study of Parents and Children), which collected blood samples and dietary and sociodemographic information from 4,484 mothers in early pregnancy. The main questions to be addressed are *a*) How much does seafood contribute to prenatal blood mercury levels? and *b*) How much do other dietary sources contribute to prenatal blood mercury?

## Methods

*The ALSPAC Study.* ALSPAC aimed to enroll all pregnant women residing in Avon (a geographically defined area that includes the city of Bristol, smaller urban towns, and rural areas about 120 miles west of London, UK) with an expected delivery date between 1 April 1991 and 31 December 1992. The study enrolled 14,541 pregnant women. Its stated aims were to evaluate genetic and environmental influences on health and development, including environmental factors measured prospectively during pregnancy (ALSPAC 2013; Golding et al. 2001).

Information was collected from the mothers using four questionnaires mailed to the women during pregnancy. Dietary consumption was assessed using a food frequency questionnaire (FFQ) administered at 32 weeks gestation [see the “Your Pregnancy” questionnaire, which queried the number of occasions per time interval that the woman currently ate specific types of food, and the most frequently consumed types of milk, fats, and bread (ALSPAC 2013; see also Rogers and Emmett 1998)]. Women were offered the assistance of an interpreter or interviewer if they did not speak English or needed help to complete the questionnaire. The questions on seafood consumption (specifically, three questions concerning the frequency of consumption of white fish, oily fish, and shellfish, respectively) were partially validated by comparing responses with levels of DHA (docosahexaenoic acid) measured in maternal prenatal red blood cells, which indicated strong positive correlations (Williams et al. 2001). Ethical approval for the study was obtained from the ALSPAC Law and Ethics Committee and the local research ethics committees. (Consent for questionnaire completion was implied if the questionnaire was completed and returned to the study office—there was no compulsion to do so, and no reward was given; analyses of biological samples were carried out only with written permission.)

Collection of blood samples for trace metals. Blood samples were obtained from 4,484 women residing in two of the three Health Authority areas of the recruitment region. Samples were collected in acid-washed heparin vacutainers by midwives as early as possible in pregnancy. The sociodemographic characteristics of the women who donated samples were comparable to those of the rest of the ALSPAC study population (Taylor et al. 2013). Samples were stored for 0–4 days at 4^o^C at the collection site before being sent to the central Bristol laboratory. Samples were transported at room temperature for up to 3 hr, and stored at 4^o^C as whole blood in the original collection tubes for 18–19.5 years before analysis.

Analysis of samples. The samples were sent to the Centers for Disease Control and Prevention (CDC) for analysis of whole blood mercury, lead, selenium, and cadmium (CDC method 3009.1; unpublished information). Clotted whole blood was digested to remove all clots, before being analyzed using inductively coupled plasma dynamic reaction cell mass spectrometry (ICP-DRC-MS) (PerkinElmer 2001; Tanner and Baranov 1999; Tanner et al. 2002; Thomas 2003). The entire clotted whole blood was transferred to a digestion tube using concentrated nitric acid with the volume estimated from the weight (Taber 1965). The blood sample was heated in a microwave oven at a controlled temperature and time during which the organic matrix of the blood was digested removing the clots. ICP-DRC-MS internal standards (iridium and tellurium) were added at a constant concentration to all blanks, calibrators, and samples (at the time of 1:9 dilution of digestate) to facilitate correction for instrument noise and drift. The standard additions method of calibration was used to optimize the analytical sensitivity of the method for the whole blood samples. A recovery spike was included in each analytical run for calibration verification and as a blind quality control (QC) sample. The ICP-DRC-MS was operated in the DRC mode using oxygen when analyzing for mercury and selenium, and in standard mode when analyzing for cadmium and lead (Chang et al. 2003; Tanner and Baranov 1999; Tanner et al. 2002). Two levels of bench QC materials as well as in-house QC samples with control limits unknown to the analysts were used for daily quality control.

Limit of detection. Of the 4,484 samples, 4,131 had valid results for mercury. Three valid samples with mercury values below the assay limit of detection (LOD) (0.24 μg/L) were assigned the LOD value divided by the square root of 2. (Because the distribution of mercury exposure in people is log-normally distributed, a factor > 0.5 was deemed appropriate to reflect the likelihood that more of the results below the LOD would be closer to the LOD than zero.)

Gestational age at sample collection [known for 4,472 mothers (99.7%)] ranged from 1 to 42 weeks, with a median value of 11 weeks and mode of 10 weeks. The interquartile range (IQR) was 9–13 weeks, and 93% of the samples were collected at < 18 weeks gestation.

Dietary factors. Data for 103 food and drink items were available from the FFQ, including three items related to seafood: white fish, oily fish, and shellfish. These data were supplemented by information on frequency of prepregnancy regular alcohol consumption from a questionnaire administered in mid-pregnancy.

Sociodemographic factors. Self-reported sociodemographic factors included mother’s age at the time of the delivery, social class [graded from I (professional) to IV (semiskilled) and V (unskilled manual workers)] based on the most recent or current occupation, highest education level achieved (five ranked categories), housing tenure [home mortgaged or owned outright, rented public (council) housing, or other rented or tied accommodation], ethnic background (white or nonwhite), and parity (number of previous pregnancies resulting in a live or stillbirth).

*Statistical analyses.* We used linear regression to estimate the relative contribution of dietary factors to blood mercury levels based on *R*^2^ values. Each food item was modeled as an ordinal variable coded according to the frequency of consumption as 0, 0.5, 2, 5.5, or 10 (for no consumption or consumption once every 2 weeks, 1–3 times/week, 4–7 times/week, or more than once/day, respectively), as 0, 1, or 2 (for foods with response options of never, sometimes, or usually), 0 or 1 (for foods with no or yes response options only), or according to the actual portions consumed per week (e.g., the number of glasses of milk consumed each week).

Forward and backward stepwise regression was used to identify significant predictors of blood mercury levels using 〈 = 0.01, with and without inclusion of sociodemographic factors in the model. All models were repeated using natural log-transformed blood mercury levels as the dependent variable since the distribution was slightly skewed.

## Results

**Figure 1:**
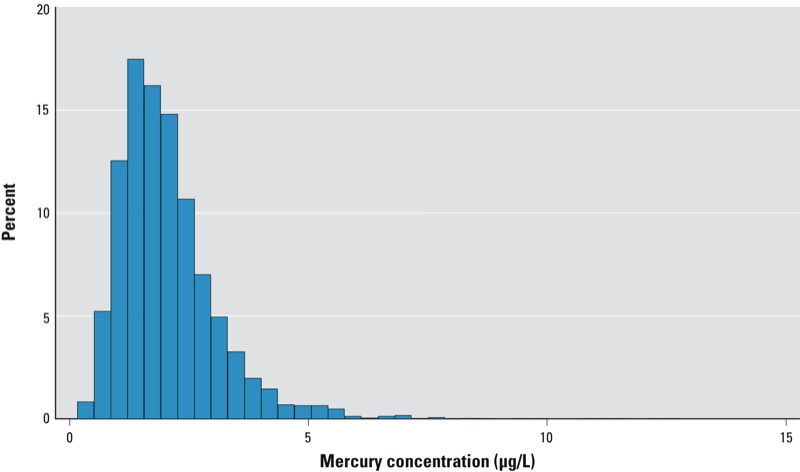
The distribution of whole blood total mercury measured in 4,134 women during pregnancy.

The frequency of seafood consumption was comparable for women with and without blood mercury data (Table 1), which suggests that the sample in the present analysis was representative of the ALSPAC study population as a whole. Blood mercury levels ranged from 0.17 to 12.8 μg/L, with a distribution that was slightly right skewed (Figure 1). The 5th, 10th, 25th, 50th, 75th, 90th, and 95th centiles were 0.81, 0.99, 1.35, 1.86, 2.52, 3.33, and 4.02 μg/L, respectively. Blood levels exceeded the 5.8 μg/L reference dose level suggested by the NRC (2000) in 38 women (0.92%).

**Table 1:** Percentage of women with a valid assay for total blood mercury among the 12,065^*a*^ ALSPAC participants with dietary data, stratified by seafood consumption patterns [*n^b^* (%)].

Seafood consumed	Never	Once/2 weeks	≥ 1 times/week^*c*^	≥ 4 times/week	*p-*Value
White fish	651 (29.44)	1,414 (29.15)	1,395 (28.86)	49 (28.65)	0.964
Oily fish	1,493 (29.26)	1,159 (29.02)	814 (28.76)	43 (30.94)	0.928
Shellfish	2,802 (28.84)	573 (29.69)	127 (32.32)	7 (25.93)	0.434
^***a***^A total of 14,541 women were enrolled in the ALSPAC Study, among whom 12,065 had dietary data during pregnancy. ^***b***^Number of women in the present analysis with a valid total blood mercury concentration (≥ LOD) who reported consumption at the level indicated, and the percentage is relative to the total number of ALSPAC participants who reported consumption at the same level. ^***c***^1–3 times per week for white fish and oily fish, ≥ 1 times a week for shellfish.					

Of the 103 dietary variables (see Supplemental Material, Table S1), blood mercury levels were positively and statistically significantly (*p* < 0.01) associated with the frequency of consumption of the following foods, listed in order of their contribution to *R*^2^: oily fish, white fish, herbal tea, alcohol, boiled rice, fresh fruit, sunflower or similar oil for frying, pasta, pure fruit juice, “health” foods, brown/granary bread, pulses, shellfish, bran cereals, wholemeal bread, salad, semiskimmed (low-fat) milk, cheese, crisp breads, olive oil on bread, organic meat, nuts, other green vegetables, organic vegetables, poultry, polyunsaturated margarine, other fat for frying, green leafy vegetables, polyunsaturated fat for frying, other organic food, goat/sheep milk, decaffeinated coffee, real coffee, oat cereals, and skimmed milk. Foods that were significant negative predictors of blood mercury levels included white bread (*R*^2^ = 3.15%) and french fries (*R*^2^ = 2.51%).

*Regression analyses of diet on untransformed blood mercury levels.* The 103 dietary variables accounted for 16.61% of the variation in blood mercury when all were included in a single linear regression model. When the model was limited to the 42 dietary variables that were positively associated with total blood mercury with *p* < 0.05 (see Supplemental Material, Table S1), the dietary variables explained 12.98% of the total variance. When we carried out a forward stepwise multivariable analysis of the 42 dietary factors only 8 were retained in the final model: consumption of alcohol (before pregnancy), white fish, oily fish, fresh fruit, brown or granary bread, frying with sunflower or similar oil, eating “health foods,” and drinking herbal teas (see model 1 in Table 2). These eight variables accounted for 11.44% of the total variance. Backwards stepwise regression produced a similar result (data not shown).

**Table 2:** Stepwise linear regression results for a model of dietary variables that were positive predictors of untransformed total blood mercury (model 1) and a model that included both positive and negative predictors of total blood mercury (model 2).^*a*^

Dietary variable^*b*^	Model 1: positive variables only		Model 2: positive and negative variables	
	β (95% CI)	*p*-Value	β (95% CI)	*p*-Value
Oily fish	0.14 (0.11, 0.18)	< 0.0001	0.14 (0.11, 0.18)	< 0.0001
White fish	0.13 (0.09, 0.17)	< 0.0001	0.14 (0.10, 0.18)	< 0.0001
Alcohol (pre­pregnancy)	0.13 (0.09, 0.17)	< 0.0001	0.12 (0.08, 0.16)	< 0.0001
Herbal tea	0.19 (0.12, 0.26)	< 0.0001	0.15 (0.08, 0.22)	< 0.0001
Health foods	0.22 (0.09, 0.35)	0.0012	0.18 (0.05, 0.31)	0.0072
Fresh fruit	0.02 (0.01, 0.03)	0.0011		
Sunflower oil for frying	0.11 (0.04, 0.19)	0.0022		
Brown/granary bread	0.13 (0.05, 0.20)	0.0005		
Meat pies or pasties			–0.08 (–0.13, –0.04)	0.0003
French fries			–0.06 (–0.09, –0.03)	0.0003
White bread			–0.13 (–0.20, –0.06)	0.0006
Sugar in tea			–0.09 (–0.14, –0.03)	0.0011
Baked beans			–0.04 (–0.07, –0.01)	0.0059
Shellfish			0.09 (0.02, 0.15)	0.0070
Milk on its own			–0.05 (–0.09, –0.01)	0.0097
^***a***^*n* = 3,432. ^***b***^Herbal tea, brown/granary bread, white bread, sunflower oil for frying, and health food consumption were modeled as binary yes/no variables; all other dietary variables were modeled as described in “Methods.”				

Forward stepwise analysis beginning with the 42 dietary factors that were positively associated with mercury (*p* < 0.05) plus 22 dietary factors that were significant negative predictors of total blood mercury selected 12 factors that accounted for 13.44% of the variance in blood mercury (Table 2). This model was based on 3,432 observations due to missing data for some of the dietary variables. Factors that were significant positive predictors were oily fish, white fish, shellfish, eating “health foods,” drinking alcohol, and drinking herbal teas, but fresh fruit and frying with sunflower or similar oil were no longer significant predictors in this model. Significant negative predictors were meat pies or pasties, french fries, baked beans, white bread, milk, and the number of spoons of sugar in tea. Backward stepwise regression produced the same model.

When we performed a linear regression that was limited to the three seafood items (white fish, oily fish, and shellfish), these items accounted for 6.98% of the total variance in blood mercury, or 42.0% (6.98%/16.61%) of the total estimated dietary contribution.

Although the information collected on the amount of alcohol consumed before conception did not include the amounts of each type of alcoholic drink, women were asked which type they preferred to drink. Mean blood mercury levels were higher among women who preferred wine (2.29 μg/L) compared with women who preferred to drink beer (1.99 μg/L) or did not drink at all (1.97 μg/L).

*Relationships between sociodemographic variables and blood mercury.* Women with the higher mean levels of total blood mercury were more likely to be older, of the higher social classes (particularly with professional or managerial occupations), more highly educated, more likely to own/have a mortgage for their own home, to be nonwhite, and expecting their first baby (all *p*-values < 0.001) (data not shown). These demographic factors explained 10.37% of the total variance in total blood mercury when all were included in a linear regression model. When the dietary factors and demographic factors were included in the same model, 16.97% of the variance was explained, compared with 13.23% for a model based on the same observations (*n* = 3,252 because of missing data for some demographic variables) that included the dietary factors only. Thus, the sociodemographic characteristics and dietary factors independently predicted blood mercury levels.

*Analyses using log-transformed mercury levels. R*^2^ estimates were increased slightly for all models when the dependent variable was ln-transformed blood mercury versus untransformed total blood mercury. Specifically, the *R*^2^ estimate based on the model of all 103 dietary variables was 19.82% (versus 16.61%); 13.58% for the model of the 42 positive dietary predictors (compared with 12.98%) (see Supplemental Material, Table S2); 13.94% for model of positive dietary predictors selected using stepwise regression (which included two additional foods, meat and semi-skimmed milk) (compared with 11.44%); 15.99% for a model of positive and negative dietary predictors selected using stepwise regression (which included meat as a new factor but not “health foods”) (see Supplemental Material, Table S2) compared with 13.44%; and 8.75% for the model that included the three seafood variables only, compared with 6.98% for the corresponding model of untransformed blood mercury. A comparison of the model with all 103 dietary variables with the model that included the three seafood variables only suggests that 44.1% of the food-related variability in log-transformed total blood mercury was explained by seafood.

*Nonlinear analyses.* Of the 12 variables in the final model, only four—oily fish, white fish, shellfish, and french fries—showed evidence of nonlinearity (see Supplemental Material, Table S3). The relationship was such that low intakes had a larger impact on blood mercury levels than expected from a linear relationship (see Supplemental Material, Table S3). Modeling these dietary factors as categorical variables increased the *R*^2^ to 16.80%, although the model had 24 degrees of freedom (df) compared with 12 df for the linear model.

## Discussion

Limiting maternal exposure to mercury to decrease potential adverse neurodevelopmental effects on the fetus has been the subject of much discussion in the medical and environmental literature (e.g., Holmes et al. 2009). The concerns have influenced public policy, and efforts to reduce maternal mercury exposure have focused on limiting seafood consumption, which has been presumed to be the chief source of exposure. However, our findings suggest that seafood accounted only for an estimated 6.98% of the variation in blood mercury levels in the pregnant women included in the analysis, who were representative of the general ALSPAC population in regard to seafood intake. This accounted for less than half of the variability in blood mercury explained by the dietary factors included in our analysis.

The estimated proportion of food-related intake associated with seafood in our study population was slightly higher than estimated from UK dietary surveys for dietary consumption of mercury over 1 week, which suggested that 25% of dietary mercury came from seafood [based on 1994 survey data (Ysart et al. 1999)] and 33% [based on 1997 data (Ysart et al. 2000)]. Their measures, however, did not take protective dietary or absorption factors into account. The higher estimate of dietary mercury consumption from seafood in the present study may reflect different forms of mercury (e.g., methylmercury) and hence different absorption rates of the different foodstuffs. Nevertheless, our findings suggest that although seafood is a component of dietary mercury exposure, it may contribute less than half of the overall mercury intake from dietary sources. Importantly, a large proportion of the blood mercury variance was not associated with any dietary variable including seafood.

Herbal teas were unexpected dietary predictors of total blood mercury in our study population. Mercury is found at relatively high levels in some folk and patent preparations (Espinoza et al. 1995; Liu et al. 2008), which are often found in health food shops in the United Kingdom, and herbal preparations such as herbal teas may have similar contaminants. However, although herbal tea consumption was a significant predictor in several models, it contributed less to the overall variance than seafood consumption because only 18% of participants reported that they drank herbal teas, whereas 88% consumed seafood.

Some dietary factors were negative predictors of total blood mercury in our study population, including white bread, whole milk, sugar, french fries, baked beans, and meat pies/pasties. Consistent with these findings, Bates et al. (2007) reported negative associations between total blood mercury and white bread, whole milk, sugar, and chips (french fries) in a study of 1,216 British adults 19–64 years of age. Although positive associations are interpreted as evidence of contamination with mercury, explanations for negative associations are less clear, but might reflect the effects of dietary constituents that limit the absorption or accelerate the elimination of mercury from the body. Alternatively, people who are more likely to eat these foods may be less likely to consume foods that are sources of dietary mercury. In this context, food items that are negative predictors of total blood mercury may be serving as a proxy indicator of low consumption of food items that are positively associated with blood mercury levels.

We have analyzed the dietary factors contributing to total blood mercury levels among pregnant women residing in Avon, an area of the United Kingdom that is largely representative of England as a whole. The analyses of maternal blood were performed in the same laboratories as the NHANES (National Health and Nutrition Examination Survey) surveys conducted in the United States (Mahaffey et al. 2004). A comparison of blood mercury concentrations of the 4,134 pregnant women who donated blood samples in 1991–1992 for the present study with those of 286 pregnant women in the 1999–2000 NHANES study suggests a marked difference. Specifically, the median value in the present UK population of pregnant women was twice that reported for the American pregnant population (1.86 vs. 0.89 μg/L, respectively), as were the 10th (0.81 vs. 0.15 μg/L) and 25th percentiles (0.99 vs. 0.38 μg/L). However, the 90th and 95th percentiles for the U.S. study (4.83 μg/L and 5.98 μg/L, respectively) were higher than in the United Kingdom (3.33 μg/L and 4.02 μg/L).

The proportion of women of childbearing age with blood mercury levels above the recommended level for adult women (5.8 μg/L) was 8% in the NHANES study (Schober et al. 2003) compared with 1.9% in a study in North Carolina (Miranda et al. 2011) and 0.9% in the present study. The difference between the high ends of the distributions of blood mercury in NHANES and ALSPAC is unlikely to be attributable to differences in the consumption of seafood, as this is less in the United States than in the United Kingdom, and the mercury levels of seafood eaten in the United Kingdom are generally higher than those of seafood eaten in the United States (Hibbeln et al. 2007).

Relatively few studies have evaluated demographic predictors of blood mercury levels. There have been reports of positive associations between blood mercury and age (Batariova et al. 2006; Caldwell et al. 2009), and maternal blood mercury levels have been associated with higher education, income, and ethnicity (Mahaffey et al. 2009; Miranda et al. 2011). Although it has been suggested that these associations may just reflect differences in the amounts and types of seafood consumed, our analysis suggested that associations with sociodemographic factors persist when adjusted for dietary factors, and vice versa.

The diet is not the only contributor to blood mercury levels. Mercury can also be absorbed from water and air, and from nondietary products such as dental amalgam fillings, beauty products, social drugs such as cigarettes and alcohol, illicit drugs, and medications. Mercury vapor in the atmosphere is absorbed mainly through the respiratory tract (Holmes et al. 2009). Once absorbed, the mercury is widely distributed to fat-rich tissues, and is readily transferred across the placenta and blood brain barriers. Major sources include refuse incineration, fossil fuel combustion, and fungicides/pesticides (Hutton and Symon 1986). It has been estimated that 9.9 tons of mercury are deposited on the United Kingdom from the atmosphere each year (41% from sources in the United Kingdom, 33% from elsewhere in Europe, and 25% from other parts of the northern hemisphere) (Lee et al. 2001).

Although there are a number of strengths to our study, including the large sample size and representativeness of the local population, there are some potential weaknesses. The blood samples assayed for mercury were stored for 18–19 years before funding was available to process them, and methods to process the clotted blood samples had to be developed *de novo* in the CDC laboratories. Although the physical integrity of the samples was maintained by ALSPAC, and the analytical methodology was verified by CDC, the age of the samples may have resulted in some degree of analytical inaccuracy.

Information on the mother’s diet was collected using a self-completed FFQ rather than weighed intakes. This method has been shown to be appropriate for estimating the intake of foods that are not eaten daily (such as seafood) (Emmett 2009), and the method has been demonstrated to provide adequate assessments of trace metal intake when compared with the duplicate diet method (Liu et al. 2010). We were not able to account for cooking methods or possible joint effects of foods and drinks consumed at the same time. Ouédraogo and Amyot (2011) have demonstrated *in vitro* that mercury bioaccessibility is reduced if the fish is fried or consumed with black tea or coffee. These findings have not been tested *in vivo* yet, but it is tempting to suggest that similar mechanisms may account for some of our protective findings. We acknowledge that the dietary measures used in this study are estimates, with a wide error component. This is likely to reduce the amount of variance explained overall. Although we believe that the errors should be similar for all dietary items, we cannot rule out the possibility that the ratio of the variance associated with seafood to the variance associated with all dietary items may be biased due to differences in the accuracy of the intake estimates for different food items.

Most studies have measured mercury in maternal hair rather than in blood on the assumption that it would give a long-term cumulative measure of fetal exposure (e.g., Davidson et al. 1998, 2008; Grandjean et al. 1997). Cord tissue mercury levels were more closely related to maternal blood levels (*r* = 0.85) than to maternal hair levels (*r* = 0.65) in a Japanese study population (Sakamoto et al. 2007), which suggests that blood mercury levels may provide a more accurate measure of fetal exposure in late pregnancy. However, maternal hair level may be a better proxy for fetal exposure early in pregnancy, corresponding to our maternal blood measures that were drawn in the first half of pregnancy. We measured total blood mercury levels rather than methylmercury levels that are likely to be the major form of mercury in fish. However, Sakamoto et al. (2007) estimated that approximately 90% of total blood mercury was methylmercury in their study population of 116 mother–infant pairs in three districts of Japan, and that correlations between total mercury and methylmercury were very high (*r* = 0.98 for maternal blood and 0.97 for umbilical cord tissue). If these estimates apply to our UK study population, total blood mercury levels are likely to be similar to blood methylmercury levels.

## Conclusions

Although we confirmed that seafood was a major dietary contributor to blood mercury levels in our study population, estimated intakes of the three seafood items evaluated in our study (white fish, oily fish, and shellfish) accounted for only 8.75% of the estimated variation in log-transformed blood mercury concentrations. Of interest are the increased mercury levels in women who drank herbal teas, as well as confirmation of a “protective effect” of foods such as french fries, white bread, and milk, and the question that is raised by these results: Where is the rest of the blood mercury coming from?

## Supplemental Material

(283 KB) PDF
