# Supplementary material for: Dietary Predictors of Maternal Prenatal Blood Mercury Levels in the ALSPAC Birth Cohort Study
Source: Environ Health Perspect. 2013 Jun 28;121(10):1214–8. doi: 10.1289/ehp.1206115 (PMC3801454; doi:10.1289/ehp.1206115)
Supplement: (283 KB) PDF [file ehp.1206115.s001.pdf]

Environmental Health Perspectives doi:10.1289/ehp.1206115

Supplemental Material

Dietary Predictors of Maternal Prenatal Blood Mercury Levels in the ALSPAC Birth Cohort Study

Jean Golding, Colin D Steer, Joseph R Hibbeln, Pauline M Emmett, Tony Lowery, and Robert Jones

Supplemental Material Table S1: Unadjusted relationship between mean total blood mercury and dietary intake dichotomized as (a) consumers and non-consumers and (b) treated as a linear relationship of frequency of consumption

	Do not consume		Consume		Difference	Linear association	
	n	Mean (SD)	n	Mean (SD)	P	R^2 (%)	P
**(i)Positive linear associations (P<0.05)**							
Oily fish	1493	1.75 (0.95)	2016	2.38 (1.12)	<0.0001	5.19	<0.0001
White fish	651	1.63 (1.02)	2858	2.22 (1.08)	<0.0001	3.48	<0.0001
Herbal tea	2803	2.01 (1.03)	454	2.51 (1.24)	<0.0001	2.96	<0.0001
Alcohol (pre-pregnancy)	293	1.81 (1.05)	3456	2.11 (1.09)	<0.0001	2.63	<0.0001
Boiled rice	834	1.85 (1.01)	2675	2.19 (1.10)	<0.0001	2.20	<0.0001
Fresh fruit	84	1.80 (0.91)	3429	2.12 (1.10)	0.0079	2.11	<0.0001
Sunflower oil for frying	1465	1.94 (1.05)	2032	2.24 (1.11)	<0.0001	1.91	<0.0001
Pasta	697	1.88 (1.06)	2812	2.17 (1.09)	<0.0001	1.67	<0.0001
Pure fruit juice	782	1.93 (1.06)	2731	2.16 (1.10)	<0.0001	1.67	<0.0001
Health foods	3029	2.05 (1.05)	279	2.57 (1.27)	<0.0001	1.55	<0.0001
Brown/granary bread	1997	2.00 (1.07)	1509	2.26 (1.10)	<0.0001	1.35	<0.0001
Pulses	2605	2.02 (1.04)	908	2.36 (1.20)	<0.0001	1.15	<0.0001
Shellfish	2802	2.02 (1.05)	707	2.48 (1.16)	<0.0001	1.08	<0.0001
Bran cereals	1106	1.98 (1.09)	2407	2.17 (1.09)	<0.0001	0.93	<0.0001
Whole meal/wheat bread	1754	2.01 (1.05)	1752	2.21 (1.12)	<0.0001	0.91	<0.0001
Salad	350	1.80 (0.97)	3163	2.15 (1.10)	<0.0001	0.89	<0.0001
Semi-skimmed milk	1232	1.99 (1.11)	2287	2.17 (1.08)	<0.0001	0.79	<0.0001
Cheese	208	1.88 (1.20)	3301	2.13 (1.08)	0.0014	0.71	<0.0001
Crisp breads	2382	2.07 (1.08)	1131	2.20 (1.12)	0.0009	0.62	<0.0001
Olive oil or similar on bread	3159	2.09 (1.06)	338	2.34 (1.35)	0.0001	0.47	<0.0001
Organic meat	2951	2.08 (1.05)	568	2.29 (1.27)	<0.0001	0.42	0.0001
Nuts	2364	2.04 (1.04)	1149	2.26 (1.18)	<0.0001	0.42	0.0001
Other green vegetables	255	1.79 (1.13)	3258	2.14 (1.09)	<0.0001	0.38	0.0002
Organic vegetables	2406	2.05 (1.01)	1113	2.23 (1.24)	<0.0001	0.36	0.0004
Poultry	368	1.99 (1.28)	3141	2.12 (1.07)	0.0308	0.31	0.0010
Polyunsat. margarine on bread	1710	2.05 (1.09)	1787	2.17 (1.09)	0.0017	0.30	0.0011
Other fat for frying	3453	2.11 (1.09)	44	2.63 (1.29)	0.0016	0.28	0.0015
Green leafy vegetables	334	1.89 (0.94)	3179	2.13 (1.10)	0.0001	0.28	0.0016
Polyunsat. marg. for frying	3017	2.09 (1.08)	480	2.25 (1.14)	0.0024	0.27	0.0020
Other organic food	3377	2.10 (1.09)	142	2.39 (1.08)	0.0015	0.26	0.0024
Goats' or sheep's milk	3482	2.10 (1.08)	37	2.73 (1.55)	0.0005	0.24	0.0035
Decaffeinated coffee	2610	2.08 (1.07)	766	2.25 (1.16)	0.0002	0.24	0.0037
'Real' coffee (not instant)	2902	2.09 (1.06)	320	2.37 (1.34)	<0.0001	0.21	0.0071
Oat cereals	1525	2.06 (1.08)	1988	2.15 (1.10)	0.0180	0.20	0.0086

	Do not consume		Consume		Difference	Linear association	
	n	Mean (SD)	n	Mean (SD)	P	R^2 (%)	P
Skimmed milk	2804	2.08 (1.09)	715	2.22 (1.10)	0.0036	0.19	0.0089
Coffee	1657	2.13 (1.13)	1833	2.09 (1.05)	0.2875	0.18	0.0111
Other root vegetables	1300	1.98 (0.99)	2213	2.19 (1.14)	<0.0001	0.17	0.0136
Organic fruit	2572	2.07 (1.02)	947	2.21 (1.25)	0.0006	0.14	0.0268
Butter on bread	2374	2.09 (1.07)	1123	2.17 (1.15)	0.0377	0.13	0.0302
Soya milk	3453	2.10 (1.08)	66	2.43 (1.64)	0.0176	0.12	0.0404
Decaffeinated tea	3167	2.10 (1.08)	211	2.41 (1.30)	0.0001	0.11	0.0460
Eggs or quiche	493	1.93 (1.01)	3016	2.14 (1.10)	0.0001	0.11	0.0481
**(ii) Non significant (P≥0.05) linear associations**							
Milk on breakfast cereal	213	1.97 (0.96)	3306	2.12 (1.10)	0.0547	0.09	0.0758
Fat on meat	1984	2.07 (1.07)	1524	2.16 (1.12)	0.0287	0.09	0.0769
Other vegetable oil on bread	3357	2.12 (1.09)	140	1.95 (1.06)	0.0748	0.09	0.0790
Bean curd	3406	2.10 (1.07)	107	2.60 (1.49)	<0.0001	0.08	0.0844
Other milk	3449	2.10 (1.09)	70	2.45 (1.16)	0.0092	0.08	0.0851
Decaffeinated cola	2654	2.11 (1.07)	449	2.08 (1.07)	0.5894	0.07	0.1188
Biscuits	351	2.16 (1.18)	3162	2.10 (1.08)	0.3279	0.07	0.1304
Meat	347	2.17 (1.31)	3162	2.10 (1.07)	0.2683	0.06	0.1371
Milk in tea	527	2.16 (1.13)	2992	2.10 (1.08)	0.2226	0.06	0.1413
Chocolates	893	2.14 (1.13)	2620	2.10 (1.08)	0.4051	0.05	0.1644
Carrots	273	2.01 (1.21)	3240	2.12 (1.08)	0.0995	0.05	0.1682
Slices of bread	333	2.02 (1.03)	3172	2.12 (1.10)	0.0992	0.05	0.2022
Cakes or buns	517	2.06 (1.20)	2996	2.12 (1.07)	0.2910	0.04	0.2265
Low fat spread for frying	3429	2.11 (1.09)	68	1.96 (1.04)	0.2503	0.04	0.2574
Diet soft drinks	1118	2.09 (1.12)	2387	2.12 (1.08)	0.4278	0.03	0.2691
Offal	3139	2.12 (1.11)	370	2.05 (0.92)	0.2597	0.03	0.2923
Sterilized milk	3369	2.11 (1.10)	150	2.10 (1.00)	0.9271	0.03	0.2936
Tahini (sesame seed paste)	3408	2.10 (1.08)	105	2.43 (1.30)	0.0020	0.03	0.3292
Pizza	1506	2.09 (1.10)	2003	2.12 (1.09)	0.4400	0.03	0.3303
Chapattis	3441	2.11 (1.09)	65	2.22 (1.40)	0.3950	0.02	0.3924
Milky drink	1222	2.08 (1.08)	2297	2.13 (1.10)	0.2251	0.02	0.3943
Soya meat substitutes	3236	2.09 (1.07)	277	2.30 (1.32)	0.0033	0.01	0.5101
Boiled or baked potatoes	108	2.04 (1.45)	3401	2.11 (1.08)	0.4809	0.01	0.5250
Tinned fruit juice	2851	2.12 (1.11)	662	2.06 (0.99)	0.2319	0.01	0.5534
Dried milk	3132	2.11 (1.09)	387	2.09 (1.08)	0.7194	0.01	0.5628
Other fat on bread	3447	2.11 (1.09)	50	2.20 (1.21)	0.5760	0.01	0.5649
Hard/soft margarine for frying	3347	2.11 (1.10)	150	2.06 (1.02)	0.5483	0.01	0.5665
Low fat spread on bread	2420	2.11 (1.12)	1077	2.12 (1.03)	0.8530	0.002	0.7853
Milk in coffee	811	2.09 (1.16)	2708	2.11 (1.07)	0.6132	0.001	0.8462
Peas, sweet corn or similar	276	1.82 (1.13)	3237	2.14 (1.09)	<0.0001	0.001	0.8728
Bread with fat	212	1.98 (1.14)	3083	2.12 (1.08)	0.0558	0.001	0.8742

	Do not consume		Consume		Difference	Linear association	
	n	Mean (SD)	n	Mean (SD)	P	R^2 (%)	P
Pudding	931	2.11 (1.16)	2582	2.11 (1.07)	0.9091	0.001	0.8859
Bread	73	2.10 (1.00)	3433	2.11 (1.09)	0.9167	0.0003	0.9201
Takeaways	1092	2.05 (1.07)	2415	2.14 (1.10)	0.0185	0.0001	0.9509
**(iii) Negative linear association (P<0.05)**							
White bread	1453	2.34 (1.15)	2053	1.95 (1.02)	<0.0001	3.15	<0.0001
Chips	679	2.44 (1.28)	2830	2.03 (1.03)	<0.0001	2.51	<0.0001
Roast potatoes	1067	2.30 (1.16)	2442	2.03 (1.05)	<0.0001	1.73	<0.0001
Sugar in tea	2621	2.18 (1.09)	897	1.92 (1.09)	<0.0001	1.59	<0.0001
Meat pies or pasties	1580	2.25 (1.16)	1929	2.00 (1.02)	<0.0001	1.36	<0.0001
Sausages or burgers	1398	2.27 (1.24)	2111	2.00 (0.97)	<0.0001	1.22	<0.0001
Sugar in coffee	2580	2.16 (1.10)	938	1.96 (1.06)	<0.0001	1.11	<0.0001
Full fat milk	1434	2.20 (1.11)	2085	2.05 (1.07)	<0.0001	0.75	<0.0001
Caffeine	212	2.35 (1.21)	3210	2.10 (1.09)	0.0015	0.69	<0.0001
Baked beans	582	2.19 (1.18)	2931	2.10 (1.07)	0.0710	0.69	<0.0001
Hard/soft margarine on bread	2948	2.15 (1.11)	549	1.90 (1.00)	<0.0001	0.66	<0.0001
Cola	2078	2.22 (1.11)	1382	1.95 (1.05)	<0.0001	0.65	<0.0001
Crisps	796	2.24 (1.16)	2713	2.07 (1.07)	0.0002	0.59	<0.0001
Milk to drink on its own	1012	2.19 (1.06)	2507	2.08 (1.10)	0.0043	0.55	<0.0001
Tea	623	2.13 (1.11)	2861	2.11 (1.09)	0.6660	0.53	<0.0001
Butter for frying	2703	2.16 (1.08)	794	1.97 (1.12)	<0.0001	0.51	<0.0001
Other vegetable oil for frying	2402	2.16 (1.11)	1095	2.00 (1.05)	0.0001	0.41	0.0001
Fried food	1750	2.20 (1.12)	1759	2.02 (1.06)	<0.0001	0.40	0.0002
Chocolate bars	608	2.23 (1.21)	2905	2.09 (1.07)	0.0044	0.40	0.0002
Milk as pudding	1152	2.15 (1.18)	2367	2.09 (1.05)	0.1008	0.36	0.0004
Other cereals	1113	2.24 (1.16)	2400	2.05 (1.05)	<0.0001	0.31	0.0010
Sweets	1451	2.17 (1.09)	2062	2.07 (1.09)	0.0037	0.19	0.0088

N.B. Only 98 of the 103 variables are listed above. The five exclusions concerned continuous and categorical versions of the same data relating to specific drinks. In general, the continuous version performed better except for herbal teas where the categorical version (never, sometimes, often) performed marginally better than cups of herbal tea.

Some foods showed evidence of non-linearity most notably peas, sweetcorn or similar vegetables – they performed better dichotomized (r^2 = 0.64%) than using frequency of consumption (r^2 = 0.001%).

Supplemental Material Table S2: Linear regression of ln blood Hg levels for models including only dietary variables with positive associations (Model 1) and both positive and negative associations to total mercury (Model 2) as defined in Table 2 (N=3432)

Dietary variable	Model 1 Positive variables only		Model 2 Positive and negative variables	
	B (95% CI)	p	B (95% CI)	p
White fish	0.073 (0.056, 0.089)	$2.5x10^{-18}$	0.079 (0.062, 0.095)	$2.4x10^{-21}$
Oily fish	0.068 (0.051, 0.085)	$1.4x10^{-14}$	0.068 (0.051, 0.085)	$1.1x10^{-14}$
Alcohol (pre-pregnancy)	0.058 (0.040, 0.076)	$2.4x10^{-10}$	0.053 (0.035, 0.071)	$5.5x10^{-9}$
Sugar in tea			-0.054 (-0.077, -0.031)	$2.7x10^{-6}$
White bread [a]			-0.064 (-0.097, -0.032)	0.0001
Chips			-0.025 (-0.038, -0.012)	0.0002
Herbal tea [a]	0.075 (0.044, 0.106)	$1.9x10^{-6}$	0.057 (0.026, 0.087)	0.0003
Sunflower oil for frying [a]	0.071 (0.039, 0.103)	$1.3x10^{-5}$		
Brown/granary bread [a]	0.067 (0.036, 0.098)	$2.6x10^{-5}$		
Fresh fruit	0.008 (0.004, 0.013)	0.0005		
Pies or pasties			-0.033 (-0.052, -0.014)	0.0009
Milk on its own [b]			-0.030 (-0.048, -0.012)	0.0009
Baked beans			-0.023 (-0.036, -0.009)	0.0011
Shellfish			0.036 (0.008, 0.065)	0.0131
Health foods [a,c]	0.083 (0.025, 0.141)	0.0050	0.067 (0.010, 0.125)	0.0217
R^2 (%)	13.58		15.77	

[a]Binary yes/no variables

[b] Categorical variable with ordinal estimates of consumption frequency

[c] Separate stepwise analyses of ln Hg levels substituted meat intake for this variable in the final model.

Supplemental Material Table S3: Non-linearity of dietary variables predicting mercury levels (N=3432)

Dietary variable	Portions pw[a]	B (95% CI)	Deviation p
**Non-linear variables**			
White fish	<1	0.30 (0.20, 0.40)	<0.0001
	1-3	0.49 (0.38, 0.59)	
	4-7	0.55 (0.23, 0.86)	
	>7	0.52 (-1.07, 2.11)	
Oily fish	<1	0.29 (0.20, 0.37)	<0.0001
	1-3	0.41 (0.31, 0.51)	
	4-7	0.44 (0.12, 0.77)	
	>7	-0.17 (-1.77, 1.43)	
Shellfish	<1	0.19 (0.10, 0.28)	0.0031
	1-3	0.15 (-0.03, 0.33)	
	4-7	-0.08 (-1.05, 0.90)	
	>7	-0.17 (-1.29, 0.95)	
Chips	<1	-0.18 (-0.28, -0.09)	0.0006
	1-3	-0.26 (-0.36, -0.16)	
	4-7	-0.31 (-0.52, -0.10)	
	>7	-0.05 (-0.65, 0.56)	

[a] Reference category ‘zero portions per week’

Deviation p is the p value for the deviation from linearity.
